# Comparative sequence- and structure-inspired drug design for PilF protein of *Neisseria meningitidis*

**DOI:** 10.1186/s40246-015-0027-1

**Published:** 2015-04-19

**Authors:** Abijeet Singh Mehta, Kirti Snigdha, M Sharada Potukuchi, Panagiotis A Tsonis

**Affiliations:** Department of Biology, University of Dayton, Dayton, OH USA; School of Biotechnology, Shri Mata Vaishno Devi University, Katra, J&K India

**Keywords:** Type IV pilus assembly protein (PilF), Homology modeling, *In silico* drug designing, Molecular docking, ADME analysis

## Abstract

Serogroup A of *Neisseria meningitidis* is the organism responsible for causing epidemic diseases in developing countries by a pilus-mediated adhesion to human brain endothelial cells. Type IV pilus assembly protein (PilF) associated with bacterial adhesion, aggregation, invasion, host cell signaling, surface motility, and natural transformation can be considered as a candidate for effective anti-meningococcal drug development. Since the crystal structure of PilF was not available, in the present study, it was modeled after the Z2491 strain (CAM09255.1) using crystal structure of chain A of *Vibrio cholerae* putative Ntpase EpsE (Protein Data Bank (PDB) ID: 1P9R) and then we based this analysis on sequence comparisons and structural similarity using *in silico* methods and docking processes, to design a suitable inhibitor molecule. The ligand 3-{(4S)-5-{[(1R)-1-cyclohexylethyl]amino}-4-[(5S)-5-(prop-2-en-1-yl) cyclopent-1-en-1-yl]-1,4-dihydro-7H-pyrrolo[2,3-d] pyrimidin-7-yl}-1,2-dideoxy-b-L-erythro-hex-1-en-3-ulofuranosyl binds to the protein with a binding energy of −8.10 kcal and showed a drug likeness of 0.952 with no predicted health hazard. It can be utilized as a potent inhibitor of *N. meningitidis* pilus-mediated adhesion to human brain endothelial cells preventing meningeal colonization.

## Introduction

*Neisseria meningitidis* or *meningococcus* known for causing meningitis and other forms of meningococcal diseases is one of the prime causes of child mortality in industrialized countries and epidemics in Asian and African countries. *N. meningitidis* is an aerobic, gram-negative, non-endospore forming, non-motile (although has pilus), coccal parasitic bacterium [1]. Most strangely, this bacterium thrives in the human throat commensally and colonizes in a distinct number of populations as a healthy carrier [2]. Of the five common serogroups (A, B, C, Y, W135) responsible for about 90% of infections, serogroups A, B, and C account for most cases throughout the world, with the serogroup A strain responsible for epidemic diseases in developing countries [2,3]. The strain Z2491 [GenBank: CAM09255.1] is included under serogroup A.

*N. meningitidis* has shown widespread resistance to the currently used drugs and thus demands an advanced drug strategy to combat it. New drug targets will be helpful in the discovery of more diverse and potent drugs. Mainly, enzymes involved in macromolecular and metabolite synthesis appear to be promising potential targets, few of which have already been validated in many microorganisms. The two component signal transduction systems of bacteria which enable it to sense, respond, and adapt to their environmental or intracellular state variations are quite essential for the growth and survival in adverse environmental conditions. This system is ubiquitous in bacteria and has been reported to be involved in virulence. The translocation of proteins that is associated with the virulence factors across the outer membrane is supported by the type II secretion pathway. For the pathogen-specific type II secretion pathway, five virulent factors i.e., type IV pilus assembly protein (PilF), pilus assembly protein (PilG), twitching motility protein1 (PilT-1), twitching motility protein2 (PilT-2), and competence protein (comA) are found to be involved. However, the major components of the secretion pathway are type IV pilus assembly proteins. Type IV pili are responsible for bacterial adhesion, aggregation, invasion, host cell signaling, surface motility, and natural transformation [4]. As reported by Coureuil et al. [5] the initiation of signaling cascades by *meningococcus* follows the pilus-mediated adhesion to human brain endothelial cells that eventually leads to opening of intercellular junctions and allows meningeal colonization. Hence, for the development of an effective anti-meningococcal drug, type IV pilus assembly protein (PilF) can serve as a potential target [3].

Since the crystal structure of PilF was not available, the present study was conducted to model after the Z2491 strain using the crystal structure of chain A of *Vibrio cholerae* Putative Ntpase EpsE [PDB: 1P9R] as a template and then using *in silico* methods and docking processes. Thus, a suitable inhibitor molecule has been designed. Further, this molecule has been checked for its possible health effects and drug likeness using bioinformatic tools.

## Methods

### Sequence alignment and structure prediction

The FASTA sequence of the target protein type IV pilus assembly protein (PilF) of the *Neisseria meningitidis* Z2491 strain [GenBank: CAM09255.1] was retrieved from the NCBI Entrez sequence search [http://www.ncbi.nlm.nih.gov]. Following the BLASTp run [http://blast.ncbi.nlm.nih.gov/Blast.cgi] against the Protein Data Bank database [http://www.rcsb.org/pdb/], the crystal structure of chain A of *Vibrio cholerae* Putative Ntpase EpsE [PDB: 1P9R] was selected as a template sequence. The 3D structure of the query protein was predicted by the automated homology modeling program, Modeller 9.10 [6]. For modeling, the template and target sequences were carefully aligned to remove potential alignment errors. Structural models were visualized by Swiss Pdb Viewer (SPDBV) version v 4.0.4 [http://www.expasy/spdbv.org]. Validation of the model was performed by the Ramachandran plot analysis [7], using SPDBV and the online tool, PROCHECK [8] [http://nihserver.mbi.ucla.edu/SAVES/]. Further evaluation was carried out using ProSA-web [https://prosa.services.came.sbg.ac.at/prosa.php] and Verify_3D [http://nihserver.mbi.ucla.edu/SAVES/].

### Active site, lead prediction, and ligand building

The active domains of the developed model were determined using the software LIGSITE^*csc*^ [9] [http://projects.biotec.tu-dresden.de/pocket/]. LIGSITE^*csc*^ is a web server for the automatic identification of pockets on a protein surface using the Connolly surface and the degree of conservation. From the study of interaction between different chemical components of ligands with type II secretion system in bacteria, a potential lead molecule was determined and modified using ChemSketch version 12.01 [http://www.acdlabs.com], which was then integrated with a modeled protein structure using the Hex 6.3 [http://hex.loria.fr/]. *Hex* is an interactive protein docking and molecular superposition program. The protein-lead complex was then used to develop the ligand using the software LigBuilder version 1.3 [http://www.chem.ac.ru/Chemistry/Soft/LIGBUILD.en.html], which is a general-purpose program package written for a structure-based drug-design procedure. Based on the 3D structure of the target protein, it can automatically build ligand molecules within the binding pocket.

### Autodocking

The ligands developed were chosen for docking studies. The docking study was carried out using the AutoDock software version 1.5.4, which uses genetic algorithm (GA). PilF [GenBank: CAM09255.1] was loaded into AutoDock Tools (ADT) [http://autodock.scripps.edu/resources] as a receptor and made ready for docking by the addition of charges and hydrogen which any PDB file of the molecule usually does not contain, using the edit option in ADT. All the ligands were separately and individually docked with the PilF model; a grid for dock search was built for the whole molecule to find the most probable binding site in CAM09255.1 and to measure its interaction parameters with the ligands. The docking process was carried out in the default parameters of ADT.

### ADME and toxicity analysis

The absorption, digestion, metabolism, and excretion (ADME) properties are directly related to the biological effect of drugs and their fate in an organism and therefore need to be evaluated in medicinal chemistry. The ADME properties and its possible effects on health are determined using different bioinformatic tools like Molinspiration [http://www.molinspiration.com/], PASS (Prediction of Activity Spectra for Substances) [http://www.genexplain.com/pass], ACD/Labs I-Lab 2.0 [http://www.acdlabs.com/resources/ilab/index.php], and Toxtree [10].

Molinspiration offers free online services for calculation of important molecular properties (log*P*, polar surface area, number of hydrogen bond donors and acceptors, and others) as well as prediction of bioactivity score for the most important drug targets (GPCR ligands, kinase inhibitors, ion channel modulators, nuclear receptors).

## Results and discussion

### Structural evaluation of PilF protein

**Figure 1:**
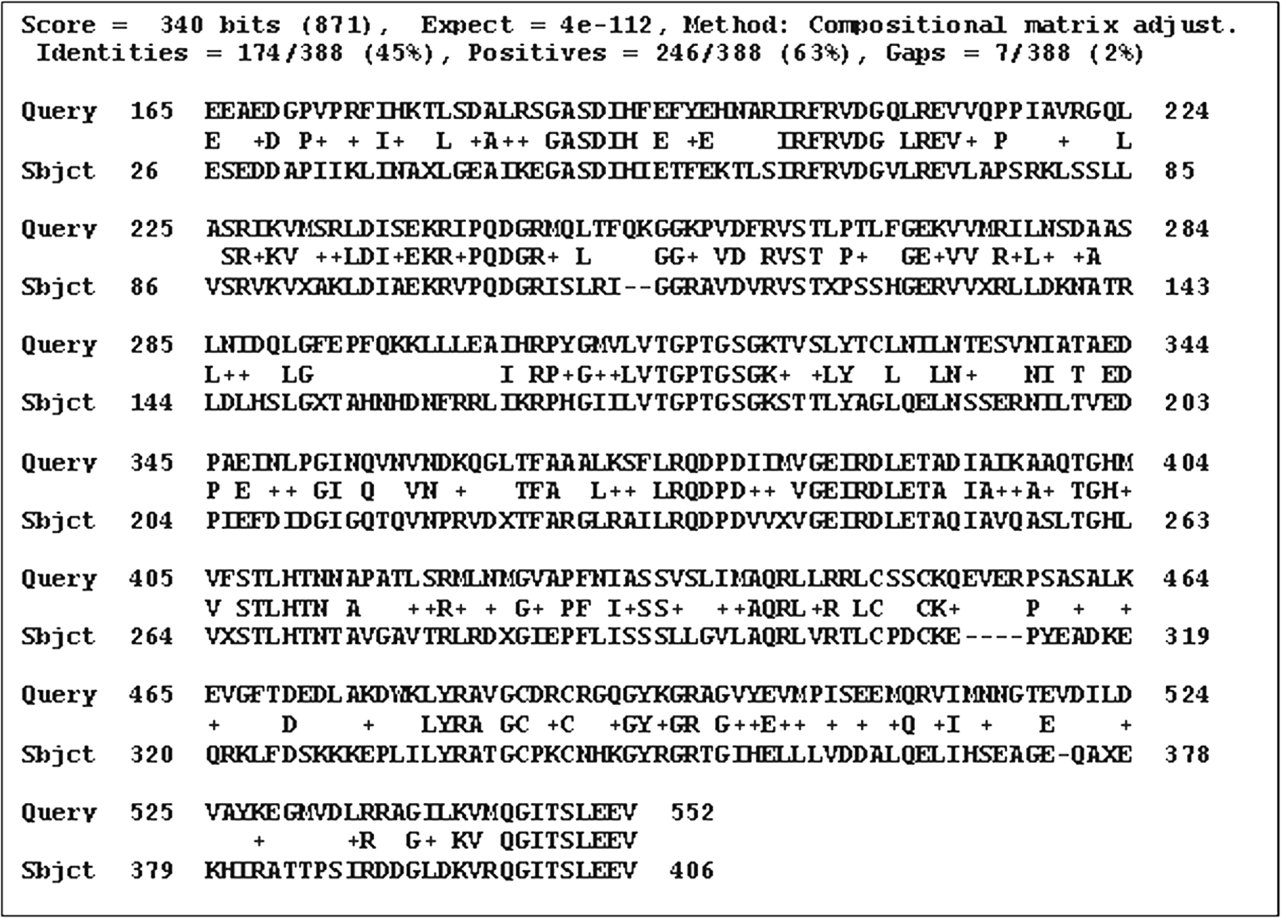
Structural evaluation of PilF protein. Secondary sequence alignment between CAM09255.1 and 1P9R by the BLASTp run [http://blast.ncbi.nlm.nih.gov/Blast.cgi] against the Protein Data Bank database [http://www.rcsb.org/pdb/]. It provided with maximum identity score and hence the crystal structure of chain A of *Vibrio cholerae* Putative Ntpase EpsE [PDB: 1P9R] was selected as a template sequence.

**Figure 2:**
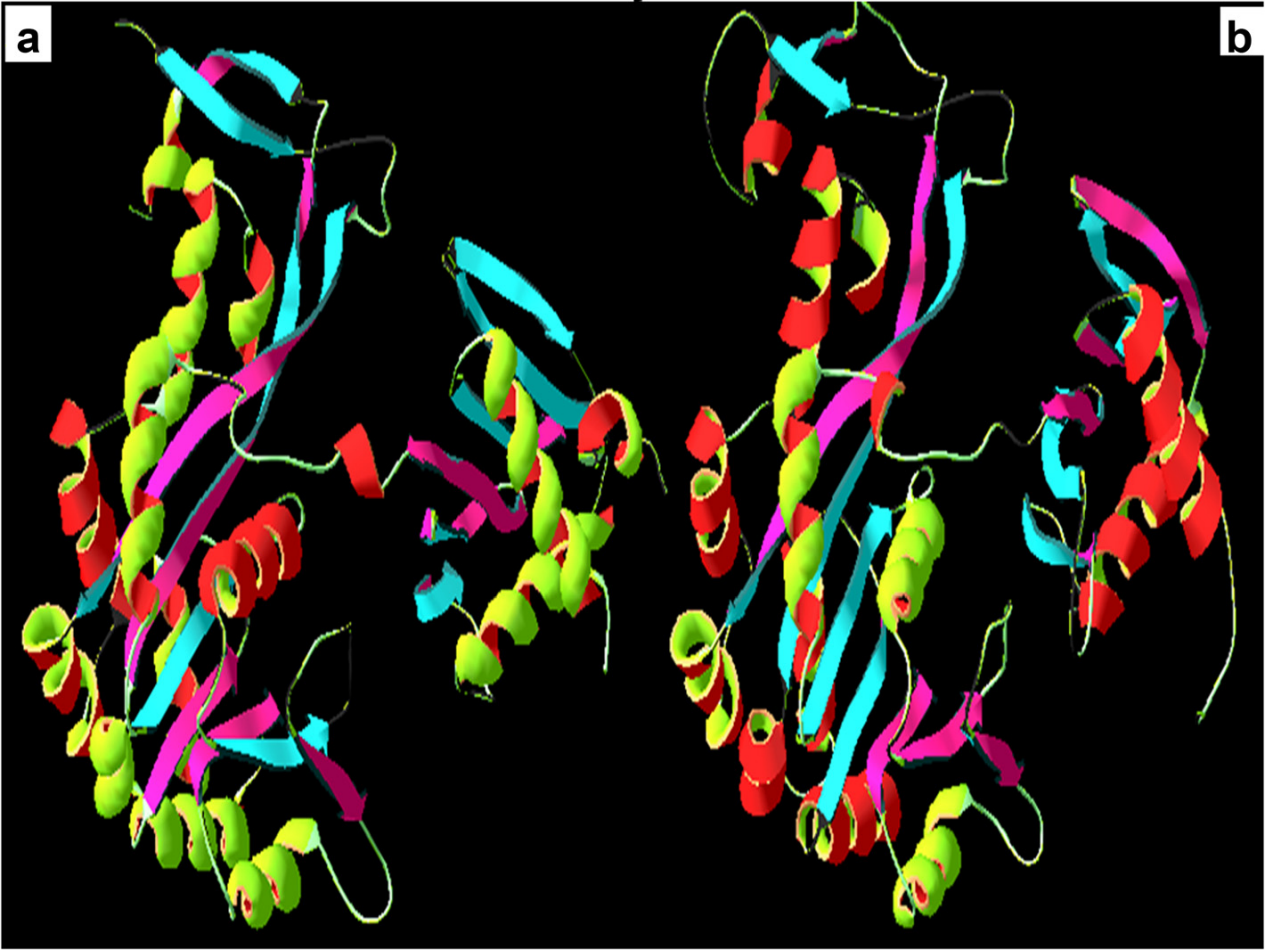
Homology modeling. **(a)** 3D structure of 1P9R and **(b)** theoretical model of PilF (CAM 09255.1) obtained after homology modeling.

**Figure 3:**
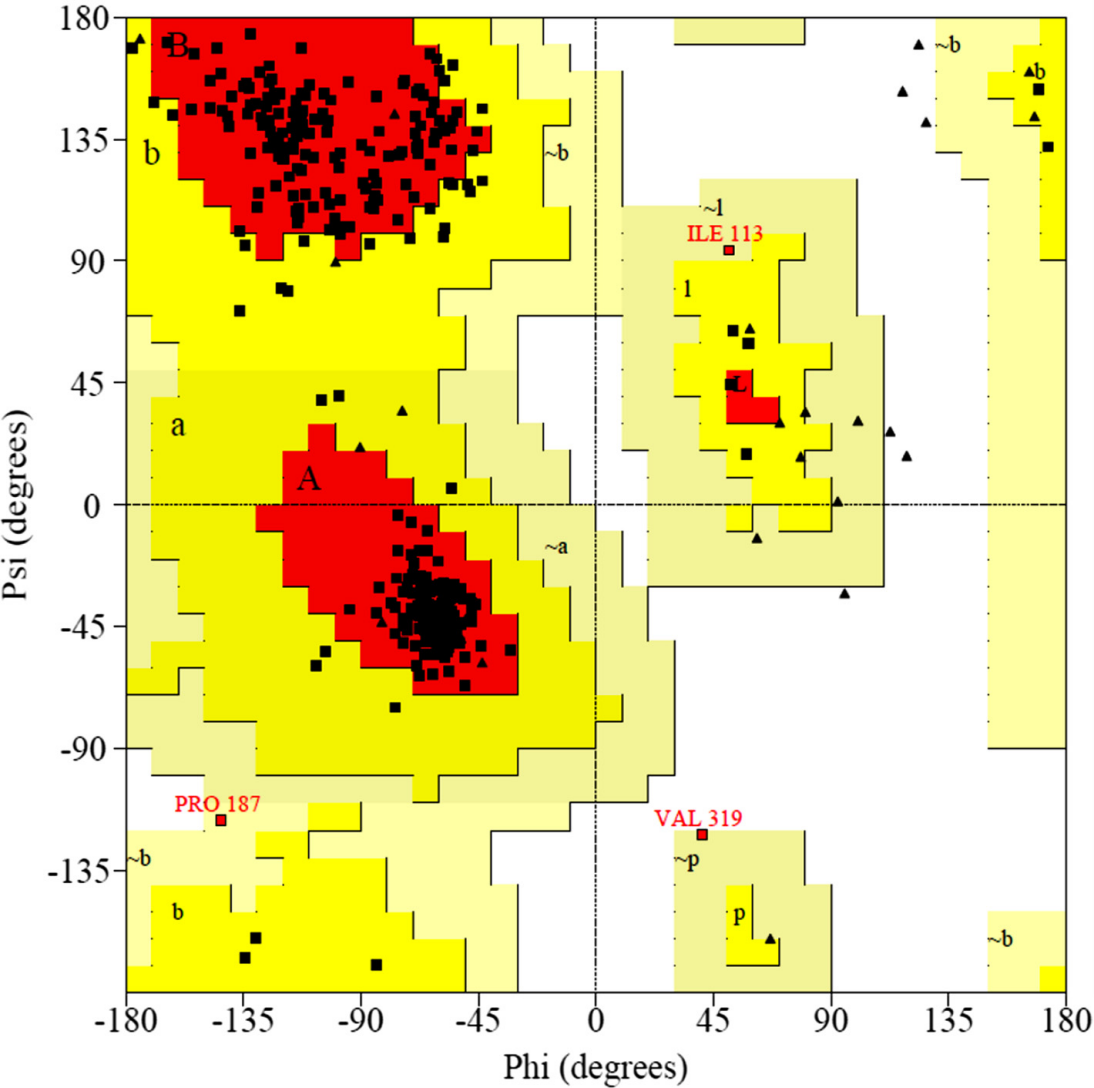
Ramachandran plot of the model. Validation of the model was done by Ramachandran plot analysis using Swiss PDB Viewer (SPDBV) version v 4.0.4 [http://www.expasy/spdbv.org]. Good overall stereochemistry is obtained for the model with 89.1% of the residue psi/phi angles falling in the most favored regions and 10.3% in the allowed region.

**Figure 4:**
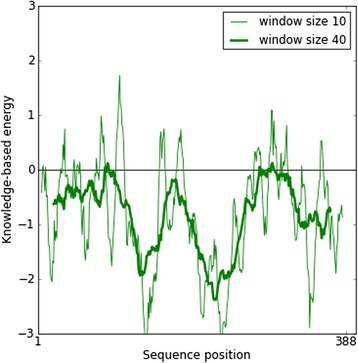
PROSA energy plot. Interaction energy per residue is calculated by program PROSA.

Structural information about the type IV pilus assembly protein (PilF) of the *Neisseria meningitidis* Z2491strain is currently unavailable. The present study reports the homology modeling and structural interaction between type IV pilus assembly protein of the *Neisseria meningitidis* Z2491 strain with an inhibitor. Chain A of *Vibrio cholerae* putative Ntpase Epse [PDB: 1P9R] having a high degree of homology with CAM09255.1 was used as a template with an identity of 45% (and conserved amino acid change of 63%). Its x-ray crystal structure has an atomic resolution of 2.50 Å and an R value of 0.217. The secondary structure alignment obtained between the query and template sequence is shown in Figure 1. Based on the Discrete Optimized Protein Energy (DOPE) score, the selected model with minimum score is shown in Figure 2b. Root mean square deviation (RMSD) value of a C alpha carbon atom between the template and predicted model was found to be 2.49A^•^ determined by using the SPDBV software. The predicted model was subjected to PROCHECK analysis to determine psi and phi torsion angles. Good overall stereochemistry is obtained for the model with 89.1% of the residue psi/phi angles falling in the most favored regions and 10.3% in the allowed region. The Ramachandran plot is shown in Figure 3. The G-factors indicating the quality of covalent and bond angle distance were −0.25 for dihedrals, 0.19 for covalent, and overall −0.07. The interaction energy per residue is also calculated by program PROSA and Verify_3D. Figure 4 displays the PROSA profile calculated for the PilF model. The comparable Ramachandran plot characteristics and G-factors confirm the quality of the predicted model (Table 1).

**Table 1:** **Validation statistics for theoretical model of PilF by PROCHECK analysis**

	**Plot statistics**	
Residues in the most favored regions [A,B,L]	304	89.1%
Residues in additional allowed regions [a,b,l,p]	35	10.3%
Residues in generously allowed regions [~a,~b,~l,~p]	2	0.6%
Residues in disallowed regions	0	0.0%
Number of non-glycine and non-proline residues	341	100.0%
Number of end-residues (exd. Gly and Pro)	2	
Number of glycine residues	28	
Number of proline residues	17	
Total number of residues	388	

### Functional site prediction, lead identification, and ligand generation

**Figure 5:**
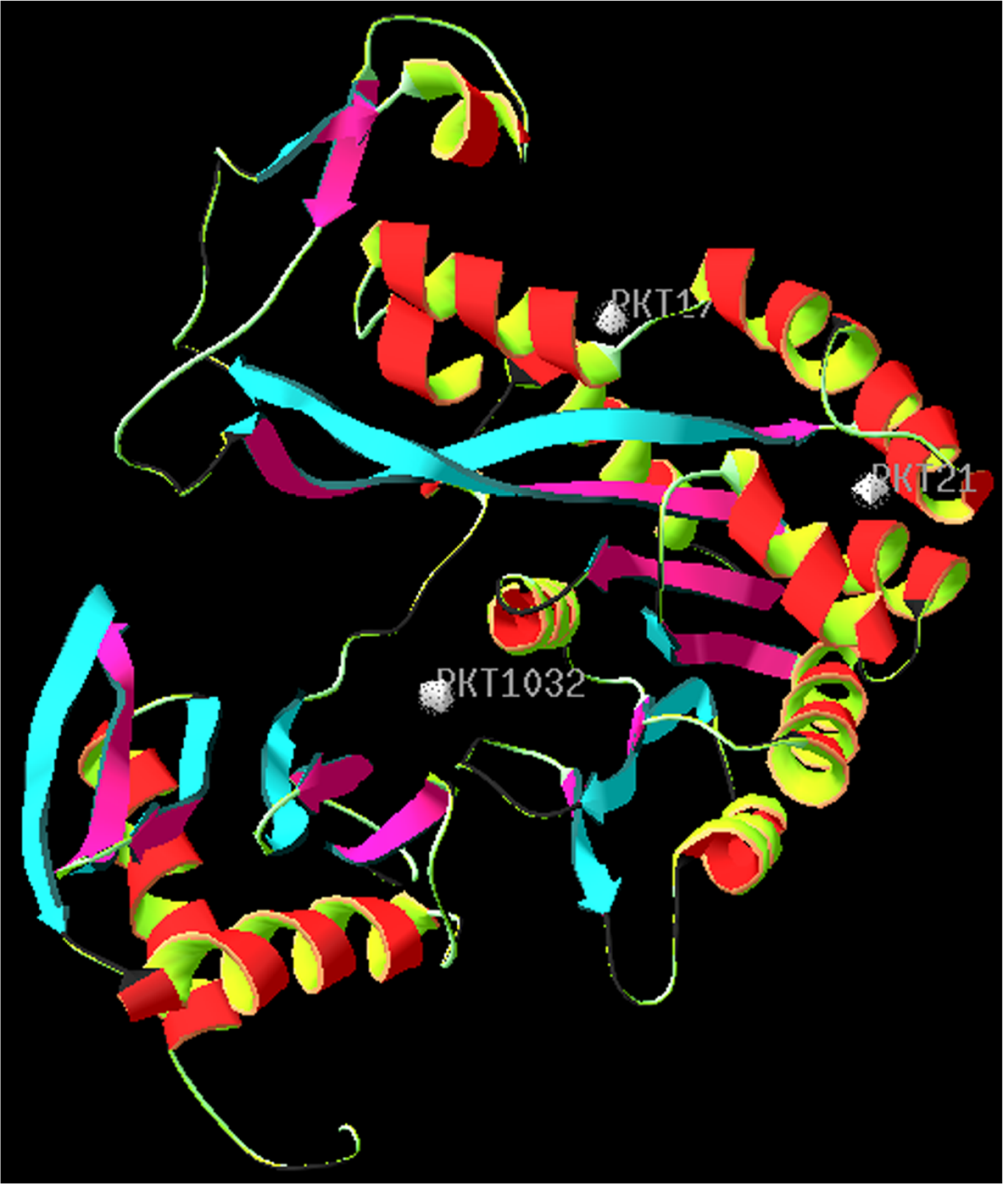
Functional site prediction using LIGSITE^*csc*^. On the basis of interaction with the surrounding atoms, out of the three pockets predicted, the pocket PKT 1032 with the best interaction result was identified as the possible functional site of the protein molecule.

**Figure 6:**
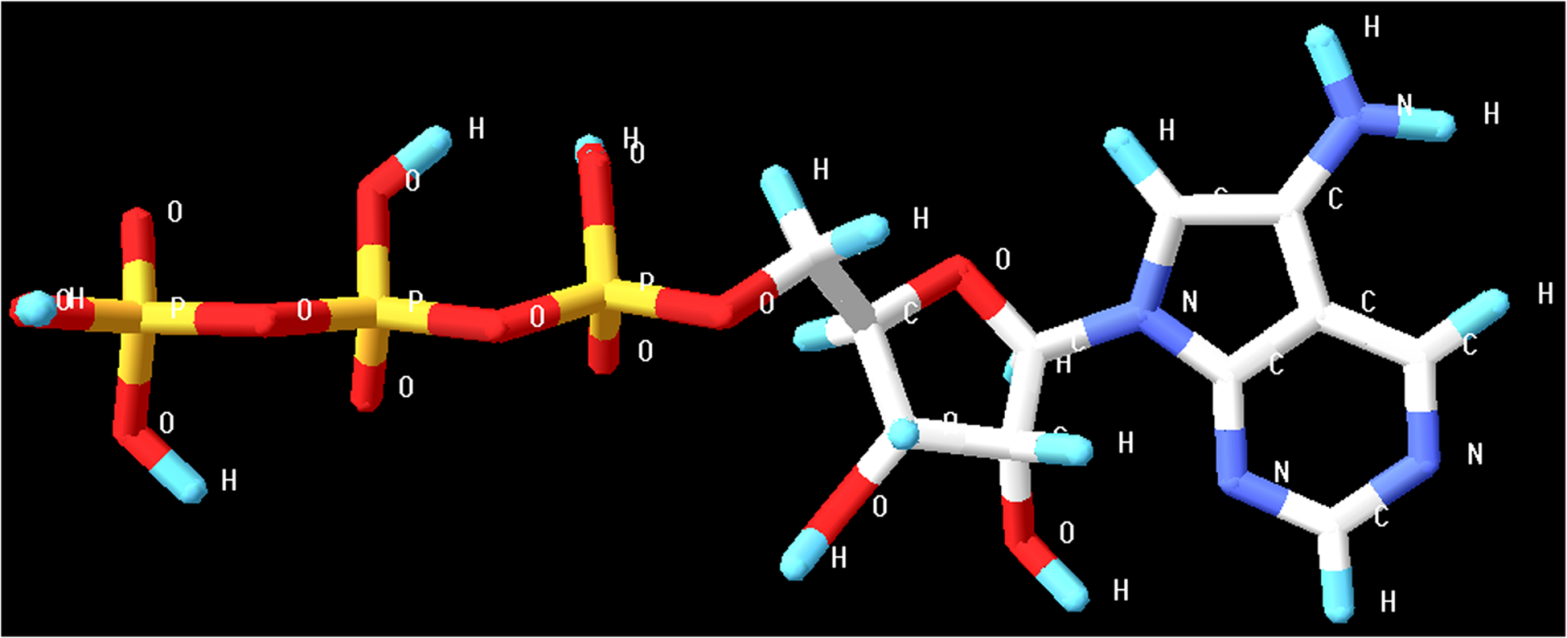
Lead introduction in protein binding site. The molecule, 7-[5-*O*(hydroxy{[hydroxyl(phosphonooxy)phosphoryl]oxy}phosphoryl)pentofuranosyl]-7*H*-pyrrolo[2,3-*d*]pyrimidin-5-amine was identified as the lead and using the Hex 6.3 [http://hex.loria.fr/] it was introduced into the protein binding site.

The software LIGSITE^*csc*^ was utilized for functional site prediction. On the basis of interaction with the surrounding atoms, out of the three pockets predicted as shown in Figure 5, the pocket PKT 1032 with the best interaction result was identified as the possible functional site of the protein molecule. The molecule 7-[5-*O*-(hydroxy{[hydroxyl(phosphonooxy)phosphoryl]oxy}phosphoryl)pentofuranosyl]-7*H*-pyrrolo[2,3-*d*]pyrimidin-5-amine (Figure 6) was identified as the lead based on the interaction study between different chemical components of ligands with the type II secretion system in bacteria. Using the Hex 6.3 [http://hex.loria.fr/], it was introduced into the protein binding site. The ten best ligands were selected for docking out of the many ligands developed by the software LigBuilder based on the Lipinski’s “Rule of 5” properties [11].

### Docking study

**Figure 7:**
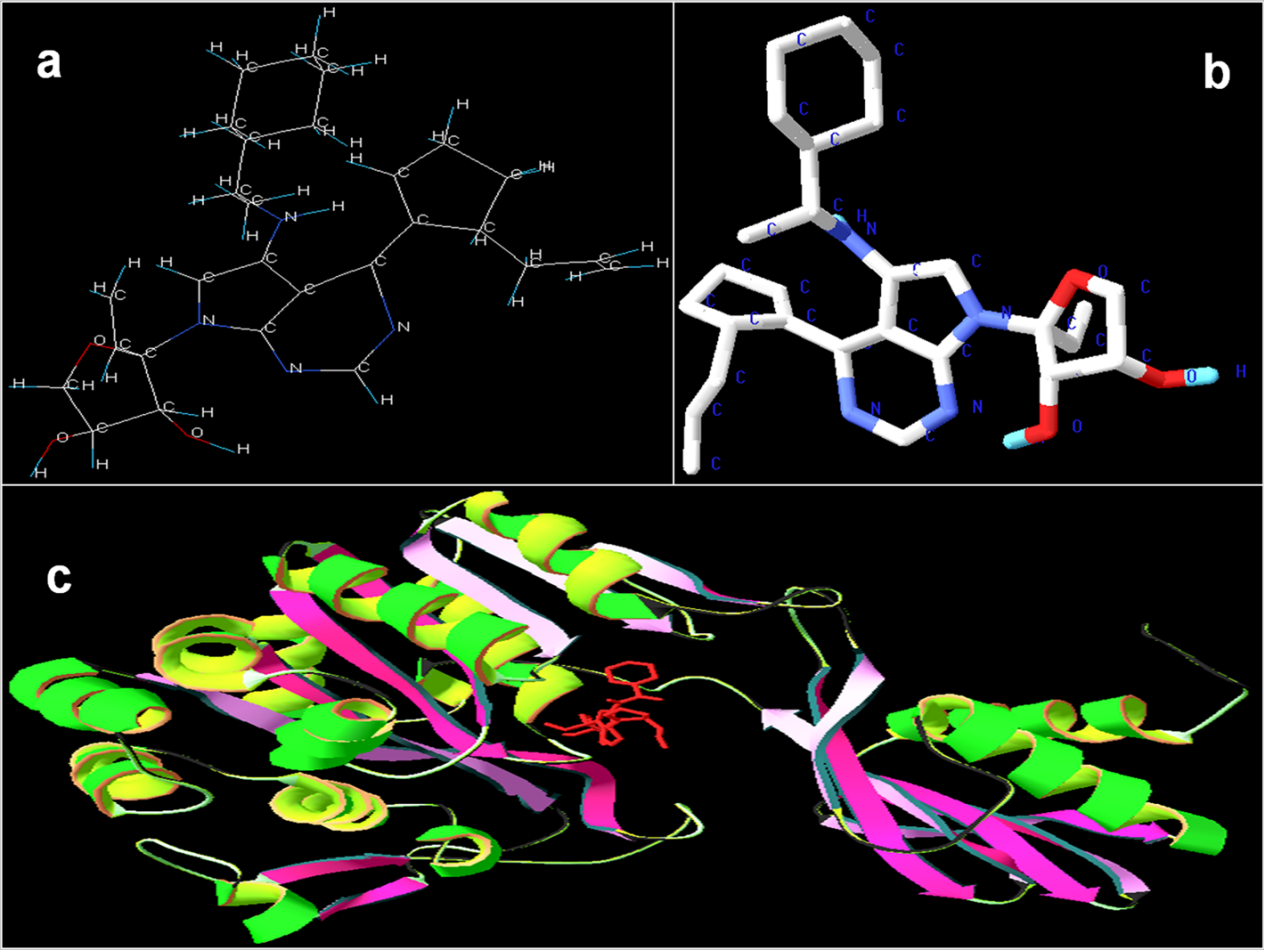
Ligand molecule and docking. Ligand molecule as build by the software LigBuilder version 1.3. **(a)** After docking using AutoDock Tools-1.5.4 for the receptor of 1P9R. **(b)** Protein-ligand complex, ligand is shown in red color. **(c)** The conformation of ligand, 3-{(4*S*)-5-{[(1*R*)-1-cyclohexylethyl]amino}-4-[(5*S*)-5-(prop-2-en-1-yl)cyclopent-1-en-1-yl]-1,4-dihydro-7*H*-pyrrolo[2,3-*d*]pyrimidin-7-yl}-1,2-dideoxy-b-L-*erythro*-hex-1-en-3-ulofuranosyl (C_28_H_44_N_4_O_3_) with PilF protein has binding energy of −8.10 kcal M^−1^ and inhibitory constant of 1.16 μM, the best docked ligand molecule.

Docking was performed using AutoDock Tools-1.5.4 for the receptor of 1P9R with all the ten ligands loaded individually into ADT to analyze ten best conformations. The study showed that the conformation of ligand, 3-{(4*S*)-5-{[(1*R*)-1-cyclohexylethyl]amino}-4-[(5*S*)-5-(prop-2-en-1-yl)cyclopent-1-en-1-yl]-1,4-dihydro-7*H*-pyrrolo[2,3-*d*]pyrimidin-7-yl}-1,2-dideoxy-b-L-*erythro*-hex-1-en-3-ulofuranosyl (C_28_H_44_N_4_O_3_) with PilF protein has a binding energy of −8.10 kcal M ^−1^ and inhibitory constant of 1.16 μM. It was concluded to be the best docked ligand molecule in comparison to other nine molecules. The ligand molecule after docking is shown in Figure 7b. The PilF protein and docked ligand in the pocket is shown in Figure 7c.

### ADME and toxicity analysis of the ligand

**Figure 8:**
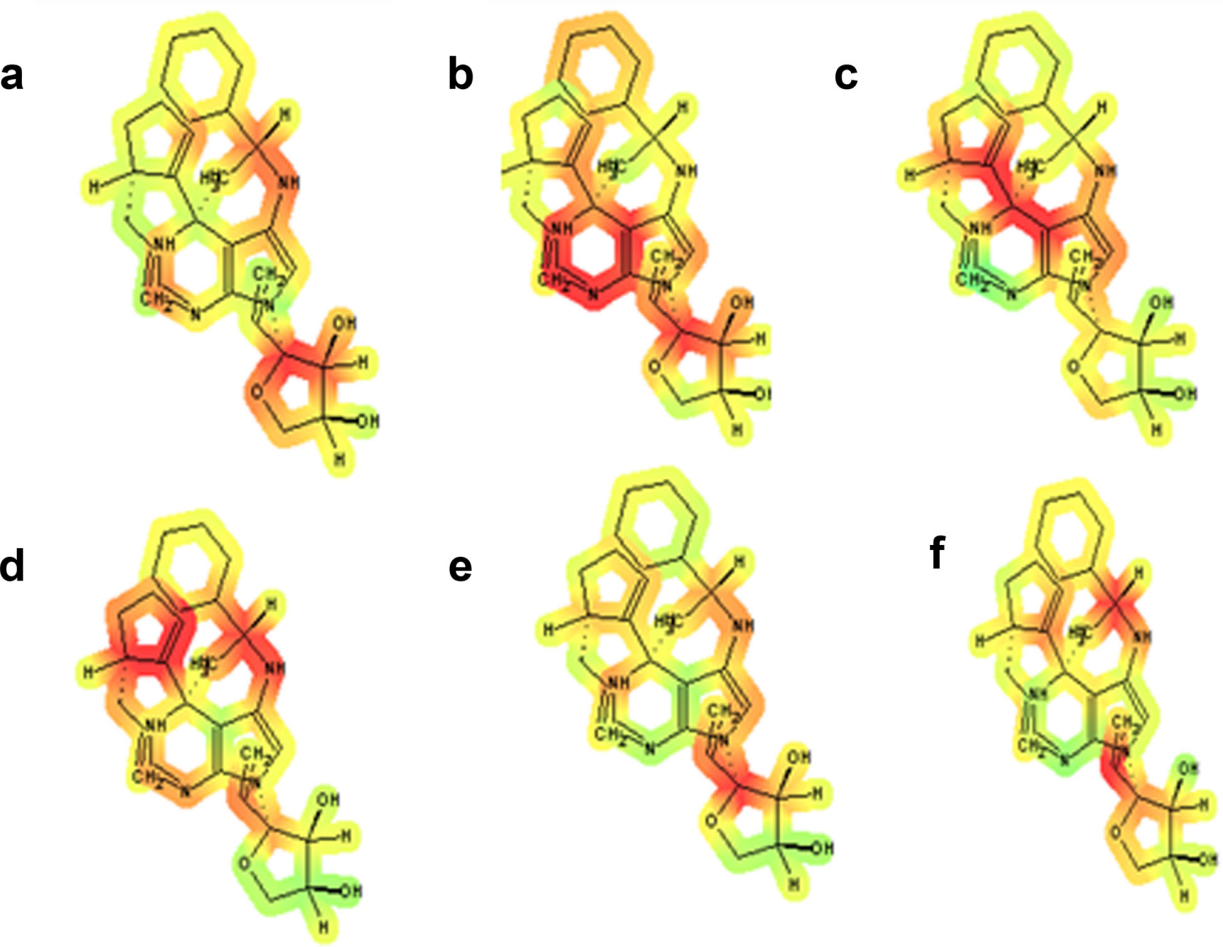
The fragmental contribution maps of ligand on **(a)** blood, **(b)** cardiovascular system, **(c)** gastrointestinal system, **(d)** kidney, **(e)** liver, and **(f)** lungs. These maps illustrate the role of individual atoms and fragments of the ligand in a color-coded manner; red color indicates a positive contribution to the toxicity whereas green color means the atom/fragment has a no relevant effect.

The pharmacokinetic features of the ligand taken into account in this work were preliminarily investigated. It is extremely advantageous if information about the ADME properties of the studied molecules could be produced in the early stages of the drug discovery process. The use of *in silico* methods to predict ADME properties is intended as a first step in this direction, and the results of such an analysis are herein reported and discussed. The molecular properties of the ligand 3-{(4*S*)-5-{[(1*R*)-1-cyclohexylethyl]amino}-4-[(5*S*)-5-(prop-2-en-1-yl)cyclopent-1-en-1-yl]-1,4-dihydro-7*H*-pyrrolo[2,3-*d*]pyrimidin-7-yl}-1,2-dideoxy-b-L-*erythro*-hex-1-en-3-ulofuranosyl as predicted by the Molinspiration property engine v2011.04 and ACD/Labs I-Lab 2.0 were found to be in accordance with the Lipinski’s “Rule of 5” properties [11], which states, that most “drug-like” molecules have log*P* <= 5, molecular weight < = 500, number of hydrogen bond acceptors <= 10, and number of hydrogen bond donors <= 5. The bioactivity of the ligand was predicted using Molinspiration Bioactivity engine v2011.06 (Tables 2 and 3). Oral bioavailability as determined by ACD/Labs I-Lab was between 30%–70%. Drug likeness of the ligand was found to be very high i.e., 0.952 calculated by PASS (http://www.ibmc.msk.ru/PASS/). Moreover, no toxic or harmful effect of the ligand over human health was predicted by PASS and Toxtree (http://toxtree.sourceforge.net/) software. Possible health effects of the ligand molecule over blood, cardiovascular system, gastrointestinal system, kidney, liver, and lungs are shown in Figure 8 and were determined by ACD/Labs I-Lab (Algorithm Version: v5.0.0.184). The software facilitates in determining specific structural toxicophores that are responsible for the organ-specific toxic effect on the following: blood, cardiovascular system, gastrointestinal system, kidney, liver, and lungs. These fragmental contribution maps illustrate the role of individual atoms and fragments of the ligand in a color-coded manner; red color indicates a positive contribution to the final predicted value or toxic action whereas green color means the atom/fragment has a negative coefficient in the regression equation i.e., unrelated to the health effects under investigation. These estimates are based on data from over 100,000 compounds from chronic, sub-chronic, and acute toxicity and carcinogenicity studies with specific to particular organ systems. Evaluation of the safety of new chemicals and pharmaceuticals requires the combination of information from various sources (e.g., *in silico, in vitro*, and *in vivo*) to provide an assessment of risk to human health and the environment [12], but *in silico* techniques can enable compounds to be deselected earlier in development, thereby limiting the need for animal testing. Previously, it has been proved that *in silico* drug design is a potent tool to screen a drug and virtually suggests its efficiency and any type of associated health hazards before evaluating the same *in vitro* [13] and/or *in vivo* [14]. Therefore, it is more likely that in the future, performing the wet lab experiments involving chemical synthesis of designed drug and testing the same *in vivo* using specific cell lines would give promising results.

**Table 2:** **Molecular properties of the ligand determined by Molinspiration property engine v2011.04**

	**Values**
Log*P*	4.07
Molecular weight	480.64
No. of hydrogen bond donors	4
No. of hydrogen bond acceptors	7
Topological polar surface area	91.042
No. of rotatable bonds	8

**Table 3:** **Molecular Bioactivities of the ligand determined by Molinspiration property engine v2011.04**

	**Values**
GPCR ligand	0.16
Ion channel modulator	−0.13
Kinase inhibitor	0.03
Nuclear receptor ligand	0.09
Protease inhibitor	0.10
Enzyme inhibitor	0.14

## Conclusions

The homology modeling of PilF protein of the *N. meningitidis* Z2491strain based on the crystal structure of chain A of *Vibrio cholerae* Putative Ntpase Epse [PDB: 1P9R] was performed. Using a structure-based drug designing method, a potent inhibitor molecule was designed. Docking studies of 3-{(4*S*)-5-{[(1*R*)-1-cyclohexylethyl]amino}-4-[(5*S*)-5-(prop-2-en-1-yl)cyclopent-1-en-1-yl]-1,4-dihydro-7*H*-pyrrolo[2,3-*d*]pyrimidin-7-yl}-1,2-dideoxy-b-L-*erythro*-hex-1-en-3-ulofuranosyl into the active site of PilF resulted in lowest binding energy signifying highest binding affinity. The *in silico* ADME and toxicity analysis confirmed its drug likeness and no predicted health hazard. Also, it can be expected that this study will be helpful in forming a base regarding the modeling of PilF protein and the development of anti-meningococcal drugs.
